# Mitochondrial Myopathy in the Oculoplastic Practice

**DOI:** 10.3390/jcm15197646

**Published:** 2026-10-02

**Authors:** Alex J. Y. Cheong, Jianbin Ding, Zhi Wei Lim, Wen Chen, Kong Bing Tan, Maryanne Chew Romero, Blanche X. H. Lim

**Affiliations:** 1 Department of Ophthalmology, National University Health System, Singapore 119228, Singapore; 2 Yong Loo Lin School of Medicine, National University of Singapore, Singapore 119077, Singapore; 3 Department of Ophthalmology, Ng Teng Fong General Hospital, Singapore 609606, Singapore; 4 Department of Pathology, National University Health System, Singapore 119077, Singapore

**Keywords:** ptosis, progressive external ophthalmoplegia, mitochondrial myopathy

## Abstract

Ptosis is a common ocular complaint in the elderly population. While most cases are often due to involutional changes in the eyelid, neurogenic, mechanical or myogenic diseases should remain as important differential diagnoses. We report a case of a 75-year-old Chinese man who presented with chronic progressive ptosis and was initially managed as presumed seronegative ocular myasthenia gravis (MG). Despite positive bedside findings suggestive of MG, serological and electrophysiological investigations were inconclusive, and there was no clinical response to pyridostigmine. The diagnosis was achieved only after an opportunistic frontalis muscle biopsy obtained during planned brow lift surgery demonstrated histopathological features supportive of a mitochondrial aetiology. This case highlights the diagnostic challenge of distinguishing mitochondrial myopathies (MM) from ocular MG, emphasises clinical features that should prompt reconsideration of the diagnosis, and illustrates how opportunistic biopsy during oculoplastic surgery may facilitate diagnosis in selected patients with persistent diagnostic uncertainty.

## 1. Introduction

Aponeurotic ptosis is the most common cause of droopy eyelids in the elderly population, described as involutional dehiscence or disinsertion of the levator aponeurosis from the tarsus [1]. Nonetheless, clinicians should also consider other differentials such as neurogenic, mechanical and myogenic diseases, which can masquerade as aponeurotic ptosis [2]. While neurogenic and neuro-muscular junction disorders are fairly straightforward to identify given their distinct clinical features and simple diagnostic tests, diagnostic evaluation for myogenic disease is less commonly performed, especially in elderly populations. In particular, mitochondrial myopathy (MM) is an often-neglected spectrum of conditions typically perceived as diseases presenting in the first three decades of life with a low reported incidence in elderly populations [3]. Yet, it is important for clinicians to recognise and workup cases of suspected myopathy, given underlying systemic associations which may be fatal [4]. We describe a case of suspected mitochondrial myopathy in an elderly Chinese male who presented to Ophthalmology with chronic progressive bilateral ptosis, whose clinical presentation and initial bedside testing raised suspicion for myasthenia gravis. We discuss the diagnostic difficulty this presentation posed, the value and limitations of opportunistic muscle biopsy performed during oculoplastic surgery, and summarise key mitochondrial disorders with ophthalmic manifestations together with their diagnostic evaluation and management.

## 2. Patient Background

A 75-year-old Chinese male was referred to Ophthalmology in June 2019 for abnormal results on diabetic retinopathy screening in the community and complaints of long-standing blurring of vision in his right eye. He had a past medical history significant for hypertension, diabetes mellitus, hyperlipidaemia, ischaemic heart disease and chronic kidney disease.

On external examination, there was brow ptosis and dermatochalasis in both eyes with right exotropia, which the patient reported to be longstanding. Visual acuity was 6/15 in the right eye and 6/6 in the left eye, and there was a significant anisometropic difference in refractive error between the two eyes. Examination of the posterior segment was unremarkable in both eyes with no pigmentary retinopathy or optic atrophy identified. There was no motility limitation, fatiguability of systemic features of myasthenia gravis (MG). Initial impression was that of strabismic amblyopia in the right eye, supported by the anisometropia and longstanding exotropia.

Over the next two years, the patient was followed up annually. In 2022, the patient was noted to have developed progressive ptosis, and was referred to the oculoplastics service for further assessment. Oculoplastics review in May 2022 revealed an enhanced Gorelick and positive Cogan’s lid twitch sign, with a positive ice-pack test. There was mild limitation of downgaze in the right eye and moderate limitation of up gaze in the left, though extraocular motility was full during his initial presentation. At this juncture, the patient reported no symptoms of weakness or fatiguability and there were no systemic features of MG noted. He also denied any diplopia.

This presentation raised clinical suspicion for MG, which prompted further diagnostic evaluation. Acetylcholine receptor and muscle-specific kinase (MuSK) antibodies were negative, and repetitive nerve stimulation (RNS) testing was normal. A trial of pyridostigmine (Mestinon) was commenced but provided no symptomatic benefit, and the patient did not report any diurnal variation in symptoms. Single-fibre electromyography (sfEMG) was performed on the orbicularis oculi muscle with borderline findings; the mean jitter (mean consecutive difference) across twelve fibre pairs was 22.08 microseconds, within the laboratory’s normal range, but two of the twelve pairs exceeded the upper limit of normal of 30 microseconds (34 and 47 microseconds respectively). Neostigmine testing was not performed as the patient was found to be bradycardic. The patient was managed as a case of possible seronegative ocular MG, with a differential of aponeurotic ptosis.

**Figure 1:**
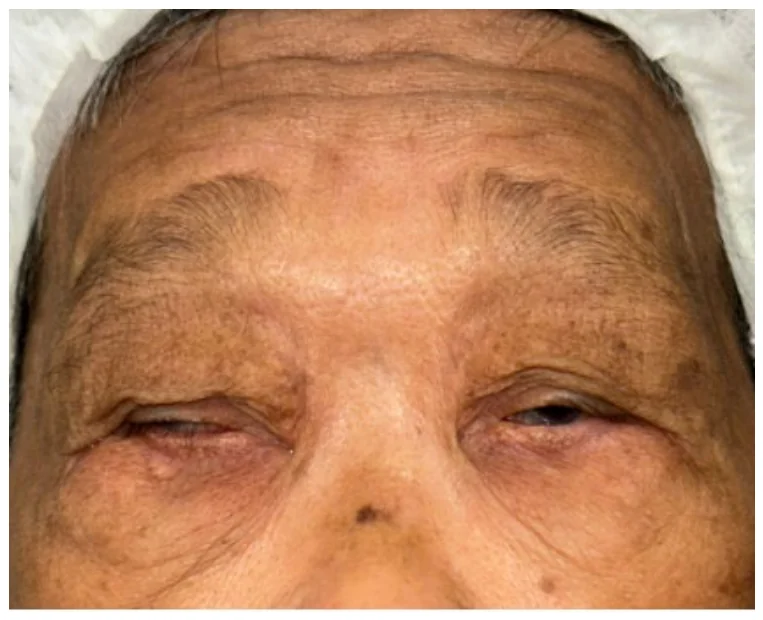
Photograph of the patient in primary gaze in the pre-operative setting.

**Figure 2:**
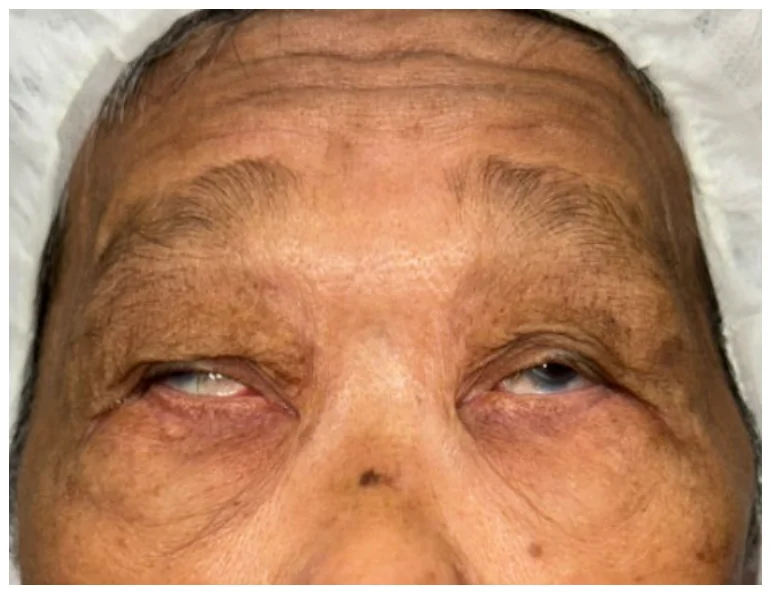
Photograph of the patient in up gaze in the pre-operative setting.

**Figure 3:**
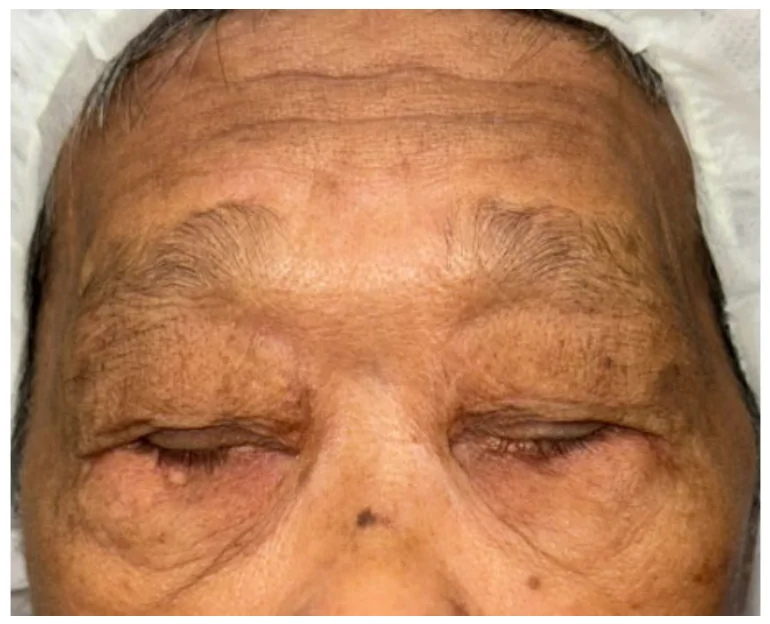
Photograph of the patient in down gaze in the pre-operative setting.

**Figure 4:**
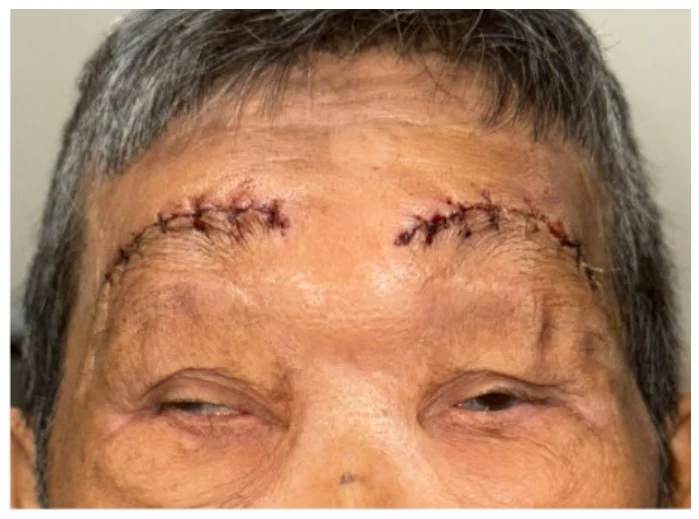
Photograph of the patient in primary gaze on post-operative day one after direct brow lift.

The patient was subsequently lost to follow-up, but he represented again in 2025 to seek surgical correction of his ptosis. At this visit, MRD1 was −1 mm in both eyes, palpebral aperture was 2 mm on the right and 3 mm on the left, and levator function was 8 mm on the right and 9 mm on the left. Pre-operative photographs of the patient are shown in Figure 1, Figure 2 and Figure 3. He was counselled for a staged procedure, with direct brow lift first followed by eyelid blepharoplasty subsequently to reduce the risk of post-operative lagophthalmos. Due to the uncertain aetiology of his presentation, an incisional biopsy of his right frontalis muscle was performed during the direct brow lift procedure. A clinical photograph of the patient one day after direct brow lift is shown in Figure 4.

The intraoperative frontalis specimen was transported fresh in saline-wet gauze and snap frozen in isopentane cooled by liquid nitrogen with optimal cutting temperature (OCT) compound. Ten-micrometre-thick sections were cut, following which Hematoxylin–Eosin stain, special stains, enzyme stains and immunohistochemical stains were performed according to laboratory validated protocol. A separate portion of muscle was fixed in formalin and processed independently for additional histochemical stains. A further portion was received in glutaraldehyde and held on standby for electron microscopy. The stains performed include Masson Trichome (MT), Gomori trichrome (GT), Periodic Acid Shiff (PAS), Phosphotungstic Acid Hematoxylin (PTAH), Cytochrome C Oxidase (COX), Sucinnate Dehydrogenase (SDH), Alkaline Phosphatase, C5-b9, CD163 and MHC1. Electron microscopy was not performed in view of cost concerns raised by the patient.

**Figure 5:**
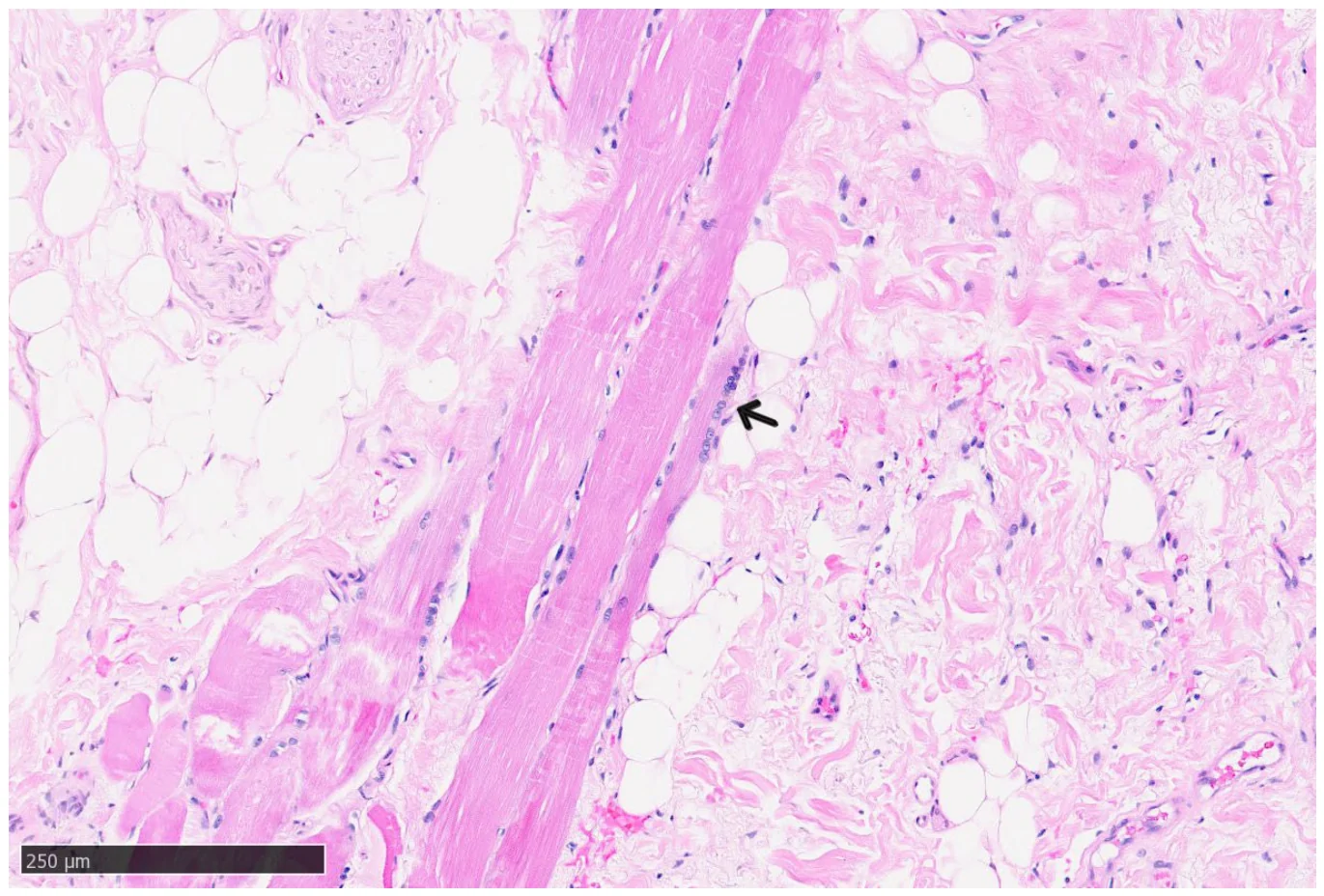
Histology image from our patient demonstrating atrophic and thin muscle fibres. Arrow indicates an atrophic multinucleated muscle fibre measuring less than 30 μm in diameter, adjacent to several relatively normal fibres. The background demonstrates fibroadipose change.

**Figure 6:**
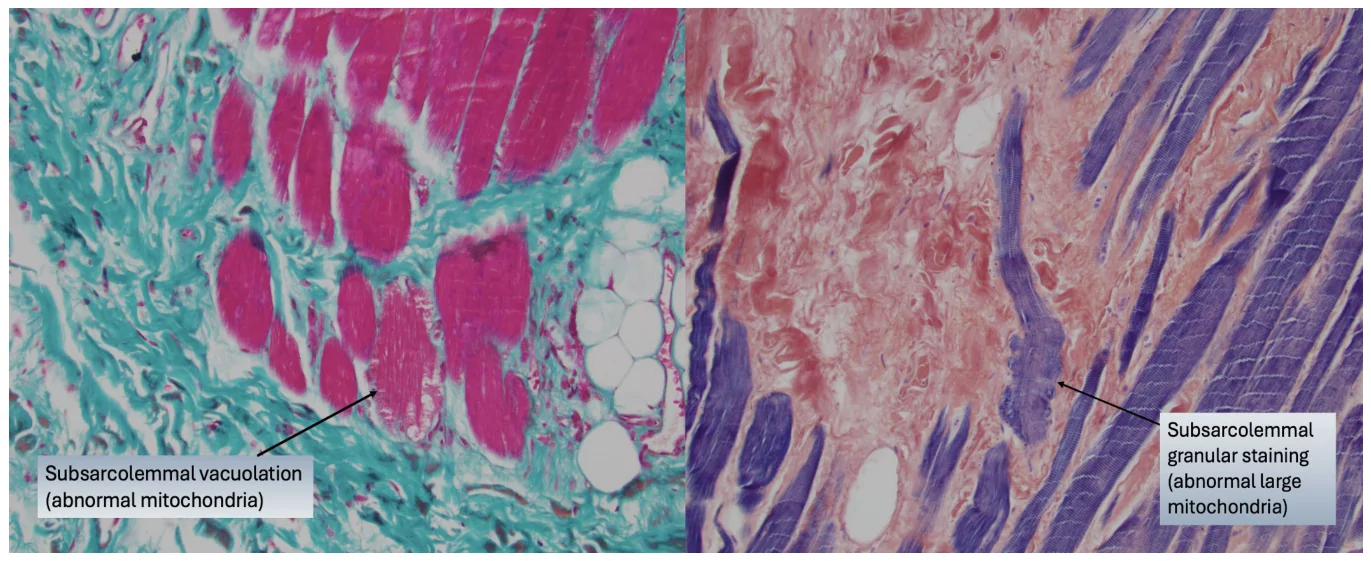
Masson Trichrome stain showing subsarcolemmal vacuolation (**left**); Phosphotungstic acid haematoxylin stain showing subsarcolemmal granular staining (**right**).

Histological analysis of the sample demonstrated scattered atrophic muscle fibres on a background of fibroadipose changes (Figure 5). Subsarcolemmal granularity was seen on PTAH with subsarcolemmal vacuolation on MT stains, suggestive of abnormal mitochondria (Figure 6). The majority of fibres did not demonstrate deficiency for COX or ragged blue appearance on SDH stain, and GT did not demonstrate ragged-red fibres (RRF). Electron microscopy was not pursued given the additional cost to the patient. Inflammatory and infective causes were not supported by the absence of significant inflammatory infiltrate on morphology or on CD163, C5b-9 and MHC class I staining, and a glycogen storage disorder was considered unlikely given the absence of significantly increased cytoplasmic staining on PAS. The patient’s histopathological findings were suggestive, but not strongly confirmatory, of an underlying mitochondrial myopathy leading to his ptosis and ophthalmoplegia.

**Figure 7:**
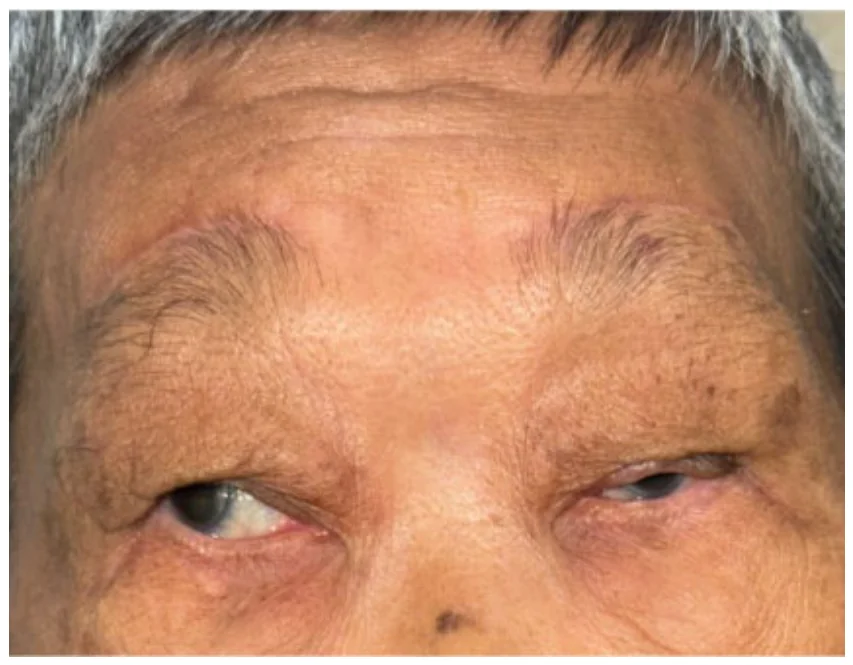
Photograph of the patient in primary gaze on post-operative month one after bilateral upper eyelid blepharoplasty.

Despite the brow lift, there was residual eyelid ptosis and hence the patient subsequently underwent bilateral upper eyelid blepharoplasty on 2 September 2025. Conservative skin resection was planned in view of the suspected mitochondrial myopathy, guided by an intraoperative pinch test to determine the extent of excess skin for excision with marking at the superior orbital rim as appropriate for this patient’s anatomy. Sufficient upper eyelid skin was preserved in accordance with the 50 by 20 mm rule. The patient experienced significant improvement in his symptoms post-operatively and the clinical photograph of the patient one month after bilateral upper eyelid blepharoplasty is shown in Figure 7. At review one year after the bilateral upper eyelid blepharoplasty, the improvement in ptosis was sustained—palpebral aperture was 7 mm on the right and 6 mm on the left, MRD1 was 1 mm on the right and 0 mm on the left, and levator function was 9 mm on the right and 6 mm on the left. There was no lagophthalmos or exposure keratopathy on examination.

In view of his pre-existing ischaemic heart disease and bradycardia, the patient underwent further cardiac evaluation: transthoracic echocardiography (August 2024) showed preserved left ventricular systolic function with an ejection fraction of 65%, myocardial perfusion imaging (September 2024) showed no evidence of ischaemia, and 24 h Holter monitoring (July 2025) demonstrated sinus bradycardia with a mean rate of 58 beats per minute, infrequent atrial and ventricular ectopic beats, and 37 brief runs of atrial tachycardia lasting three to five beats at rates of up to 89 beats per minute, without high-grade atrioventricular block. Audiology was normal and fundoscopic evaluation of the retina was unremarkable. The patient was counselled regarding genetic testing on several occasions, most recently in August 2026, and has declined each time. We summarise the timeline of events in this case in Table 1.

## 3. Discussion

This case highlights a significant diagnostic challenge when evaluating chronic, progressive ptosis in the elderly owing to overlaps in clinical presentation between various aetiologies of disease. The patient’s positive bedside tests—Cogan’s lid twitch sign, enhanced Gorelick’s sign, and a positive ice-pack test—were suggestive of ocular MG. While serological tests were normal, up to 50% of ocular MG is seronegative, keeping MG as a possible diagnosis [5]. However, there were also reasons to consider alternative diagnoses. False-positive ice-pack responses have been reported in mitochondrial myopathy, while Cogan’s lid twitch has demonstrated to be suggestive but not diagnostic of MG, and can be seen in other myopathic disorders [6,7]. The marked ptosis and external ophthalmoplegia associated with mitochondrial disease may therefore produce an MG-like clinical phenotype and complicate interpretation of bedside testing. Furthermore, despite the patient developing ophthalmoparesis during the course of his follow-up, he did not report any diplopia. In addition, his RNS test was normal and there was no clinical response to a trial of pyridostigmine. Though an abnormal sfEMG—with borderline findings in this patient—is highly sensitive for MG, its reported specificity (70%) and positive predictive value (79%) is significantly lower [8]; sfEMG abnormalities have also been reported in mitochondrial disease [9]. Table 2 highlights the clinical features that favour MG versus mitochondrial disease in our case.

Clinicians may encounter patients with presumed seronegative MG that present atypically and respond poorly to a trial of initial treatment, with equivocal results on serological and neurodiagnostic investigations. We believe this case highlights the importance of working up other differential diagnoses such as mitochondrial myopathy when managing such cases. In patients with atypical features—progressive rather than fluctuating ptosis, absence of diplopia despite motility restriction, negative serological findings and non-confirmatory RNS and sfEMG findings—clinicians should have a lower threshold to pursue muscle biopsy or genetic testing. Where possible, earlier genetic testing is the intervention most likely to shorten time-to-diagnosis in this population.

Although genetic testing is increasingly regarded as the preferred first-line diagnostic modality for mitochondrial disease, muscle biopsy continues to have an important role when molecular testing is unavailable, inconclusive, or when diagnostic uncertainty persists. COX and SDH histochemistry, together with GT staining, are key histological markers of mitochondrial myopathy. Impaired oxidative phosphorylation triggers compensatory mitochondrial proliferation, producing subsarcolemmal mitochondrial accumulation and the characteristic ragged-red fibres on GT staining. COX deficient fibres with a ragged blue appearance on SDH reflect respiratory chain dysfunction and further support mitochondrial pathology [10]. The extent of these abnormalities may vary across mitochondrial phenotypes and tissues. RRF and COX-deficient fibres are less frequently observed in mitochondrial encephalomyopathy, lactic acidosis and Stroke-like episodes (MELAS) and myoclonus epilepsy with ragged-red fibres (MERRF) due to mitochondrial DNA (mtDNA) point mutations than in CPEO due to mtDNA deletions, and are generally absent in Leber’s hereditary optic neuropathy (LHON). Conversely, COX-deficient fibres may predominate over RRFs in CPEO associated with mtDNA point mutations, single mtDNA deletions, or multiple deletions due to *POLG1* mutations. The frequency of these histopathological abnormalities appears to increase with greater levels of mtDNA heteroplasmy, demonstrating the variability of muscle biopsy findings across mitochondrial phenotypes [11].

In our patient, planned brow surgery provided an opportunity to obtain frontalis muscle tissue. Our patient’s biopsy demonstrated scattered atrophic fibres with subsarcolemmal granularity on phosphotungstic acid haematoxylin and vacuolation on Masson Trichome, with some fibroadipose change in the background. However, majority of fibres did not demonstrate deficiency for COX or ragged blue appearance on SDH. GT staining did not demonstrate ragged-red fibres. Thus, his histopathological findings were supportive, but not confirmatory of a mitochondrial disorder. Of note, the patient’s age is an additional confounding factor—clonal expansion of mitochondrial DNA deletions can occur physiologically in ageing skeletal muscle, producing atrophic, COX-deficient fibres morphologically resembling primary mitochondrial myopathy [12]. Nonetheless, the above-discussed hallmark histological features of mitochondrial disease are not always seen in patients with mitochondrial disorders and biopsy findings should be interpreted together with the clinical context and features [13]. When considering this patient’s histopathological findings alongside his clinical presentation and other investigations, the most likely diagnosis was that of mitochondrial myopathy. We suggest that his histological findings could represent a milder or earlier phenotype within the spectrum of mitochondrial disease. Patchy disease involvement and limited tissue on biopsy could also have contributed to the absence of definitive staining pattern on COX, SDH, and GT. Electron microscopy may have provided additional confirmatory evidence of abnormal mitochondria, but was not pursued in view of the additional cost to the patient. Alternative diagnoses were also considered, but were less likely to be the underlying aetiology of disease. An inflammatory or infective myopathy was considered less likely in the absence of inflammatory infiltrate on morphology or staining for CD163, C5b-9 and MHC class 1 stains. A glycogen storage disorder was also considered less likely given the absence of significant cytoplasmic glycogen accumulation on PAS staining. Where possible, electron microscopy should be considered, and genetic testing is ultimately required to identify the specific syndrome or mutation, and is necessary for prognostication, assessment of systemic risk and family counselling.

### 3.1. Spectrum of Disease in Mitochondrial Myopathy

Mitochondrial myopathy (MM) is a genetically diverse group of diseases affecting skeletal muscles. Due to dysfunction of the mitochondrial respiratory chain, oxidative phosphorylation is impaired, resulting in inadequate energy production to meet the body’s cellular demand. Patients often present with progressive weakness and episodic exercise intolerance. Individuals with MM also often have associated multi-system disease involving the heart, brain, kidneys or endocrine organs; rarely, patients may present with isolated myopathy without organ involvement [4]. MM in the paediatric population commonly presents with generalised and systemic features, while adult phenotypes are typically milder [14,15]. The estimated incidence of mitochondrial myopathy is 1 in every 4300 people, but the condition is likely to be underdiagnosed due to atypical disease manifestations. Diagnostic evaluation via pathological analysis or genetic testing can also be difficult and sometimes inaccessible [16,17].

The genetic basis of mitochondrial disease is a mutation in either mtDNA, which is maternally inherited, or nuclear DNA (nDNA), which follows Mendelian inheritance patterns. The phenotypic variability of mitochondrial disease, even within members of the same family, is explained by genetic heteroplasmy where each affected individual has a variable load of circulating wild-type and pathological mtDNA. Clinical disease manifests only when the mutation load surpasses the critical threshold of respiratory chain dysfunction [4].

Different mitochondrial disorders present with varying signs and symptoms, and the spectrum of mitochondrial myopathies can present at any point over the course of an individual’s lifespan, ranging from infancy to adulthood. Individuals affected by mitochondrial disorders commonly present with limb weakness and fatigue which may subsequently develop into respiratory failure, ophthalmoplegia, ptosis and tremors [18]. Given this patient’s symptoms presented later in life, the differentials for his condition lie at the adult end of the spectrum of disease in mitochondrial myopathy. The relevant diseases, centred on chronic progressive external ophthalmoplegia and its genetic subtypes, are summarised in Table 3 below.

Post-Viral Mitochondrial Dysfunction: There has been emerging investigation into the relationship between viral infection and mitochondrial dysfunction. Viral proteins disrupt cell membrane integrity which impairs electron transport chain function and increases oxidative stress, leading to mitochondrial damage and impaired oxidative capacity [23]. The COVID-19 pandemic has also renewed attention to viral-induced mitochondrial dysfunction, with approximately 10% of infected patients developing long COVID, characterised by persistent multisystem complications lasting weeks to months after recovery from the acute infection. Dysregulated mitochondrial function resulting from disrupted mitochondrial homeostasis during the acute infection subsequently drives chronic inflammation via persistent cytokine release and immune cell activation [24]. Viral infections aside, antiviral therapy may also result in mitochondrial dysfunction. Antiretroviral therapy with agents including emtricitabine, zidovudine, and lamivudine can induce mitochondrial toxicity through inhibition of DNA polymerase gamma and mtDNA depletion [25].

Mimics of mitochondrial disease: There are several mimics of mitochondrial disease that may manifest with similar clinical signs. Oculopharyngeal muscular dystrophy is a non-mitochondrial myopathy, with affected individuals presenting with progressive ptosis and dysphagia resulting from polyadenylate-binding nuclear protein 1 (PABPN1) mutations [26]. Myotonic dystrophy, another non-mitochondrial myopathy, may similarly manifest with ptosis and lid lag, with associated myotonia and frontal balding [27]. In paediatric or infantile patients with non-progressive disease, congenital fibrosis of the extraocular muscles (CFEOM) should be considered. Individuals with CFEOM generally have severe limitation in vertical eye movements, particularly in upgaze, with the eyes stuck in infraduction while horizontal gaze deficits are more variable [28].

### 3.2. Diagnostic Evaluation of Mitochondrial Myopathy

Diagnostic workup of suspected mitochondrial disease should begin with a detailed family and development history together with thorough clinical examination of various organ systems to identify affected organ systems. Suspicion of MM typically arises from recognition of the characteristic clinical signs, but further evaluation is often required to support the diagnosis.

Initial laboratory findings supporting the diagnosis of MM include elevated levels of creatine kinase, serum or cerebrospinal fluid (CSF) lactate, and plasma amino acids. Urine samples from affected patients may show high quantities of certain organic acids such as ethylmalonic acid and 3-methylglutaconic acid [29]. Common disorders such as thyroid eye disease and MG may present with similar clinical signs, and should be excluded through the appropriate serological and electrophysiological evaluation.

Histopathological analysis via muscle biopsy has traditionally been relied upon to study the underlying disease aetiology, as discussed in the section above. As illustrated by our patient, whose findings and proposed underlying mechanism are discussed above, these histological hallmarks are not always present or unequivocal, even where the clinical picture and milder mitochondrial changes remain compatible with an underlying mitochondrial process. A supportive but non-diagnostic biopsy therefore does not exclude the diagnosis. Of note, these histological features may not be seen in paediatric populations as degeneration of muscle tissues and accumulation of subsarcolemmal mitochondria takes time to occur. Histopathological analysis is also useful in differentiating MM from other mimickers of disease; in contrast to the abovementioned findings in MM, oculopharyngeal muscular dystrophy is characterised by basophilic rimmed vacuoles and filamentous intranuclear inclusions, while individuals with myotonic dystrophy may have muscle biopsies revealing internal nuclei, ring fibres, sarcoplasmic masses and early type I fibre atrophy [30,31]. As highlighted in our case, muscle biopsy can be considered opportunistically if the patient is already planned for other procedures. This may avoid the need for repeat procedures for biopsy or genetic testing, which can be costly, though it does not remove the need for genetic testing where the histological findings remain equivocal.

Genetic testing remains the gold standard of diagnosing mitochondrial disease. This may be performed via whole exome sequencing (WES) or whole genome sequencing (WGS). WES identifies mutations by targeting and sequencing exons, the protein-coding regions of the genome. In comparison, WGS sequences the entire genome inclusive of exons, introns and mitochondrial DNA; WGS has a higher sensitivity than WES and can identify a broader range of variant types [32]. However, WGS is understandably more expensive and generates a greater analytic burden. Thus, WES is often used as the first-line option, but as WGS has become increasingly accessible, practice is shifting towards the latter being the preferred diagnostic method where possible [33].

There are various other modalities that may be useful for evaluating a case of suspected MM, and systemic evaluation (cardiac, neurological, ophthalmological or endocrine testing) should be guided by the clinical presentation. Muscle imaging studies like magnetic resonance spectroscopy and Fourier transfer infrared spectroscopy may narrow down the specific diagnosis [34], but the clinical utility of imaging studies has been overshadowed by the rising availability of genetic and histopathological tests. In patients with neurological involvement, magnetic resonance imaging of the brain may show symmetrical signal abnormalities of the grey matter but findings are typically non-specific [35]. Functional exercise tests assist clinical evaluation of the extent and severity of disease, and can be monitored for disease progression or response to treatment. The Newcastle Mitochondrial Disease Adult Scale (NMDAS) and the International Paediatric Mitochondrial Disease Scale (IPMDS) are specific assessment scales for patients with multi-system involvement, whereas the Primary Mitochondrial Myopathy Symptom Assessment scale (PMMSA) is better suited for adults with predominantly muscle symptoms [36,37,38].

### 3.3. Management of Mitochondrial Myopathy

Management of MM is targeted towards treating disease symptoms and slowing progression of the disease, and a multidisciplinary approach is necessary. Individuals diagnosed with MM should be thoroughly evaluated for systemic involvement, and managed by the relevant specialists depending on each individual’s needs. Physical therapy, occupational therapy and speech therapy are often required to optimise motor function and preserve functional independence [39]. Catabolic states—which can be precipitated by prolonged fasting, or receiving dextrose-containing intravenous fluids commonly administered in a medical setting—should be avoided due to the potential of acute decompensation, and mitochondrial patients should be vaccinated against any preventable infections [4]. If acutely unwell, mitochondrial patients should be evaluated for derangements in routine biochemistry, glucose, transaminases and lactate, and should be monitored closely for early signs of cardiac or neurological deterioration, given their higher risk of decompensation [10].

Pharmacological therapy in MM is individualised given the phenotypic variability between different patients, and depends on each patient’s symptoms and extent of disease. Supplementation regimes have been postulated to optimised respiratory chain function and reduce oxidative stress; in patients with pigmentary retinopathy, lutein and n-acetylcysteine (NAC) may improve vision and prevent disease progression while amino acid supplementation—such as arginine, citrulline and taurine—has been effective in acute treatment prevention of metabolic stroke [40].

### 3.4. Emerging Therapies

Nutritional supplementation: Coenzyme Q10 (CoQ10) remains the most commonly used supplement in patients with MM. CoQ10 supplementation is postulated to improve mitochondrial function by restoring electron flow to the mitochondrial respiratory chain and increasing antioxidant capacity [41]. While CoQ10 use is safe and biochemically beneficial in mitochondrial myopathies, its clinical efficacy is not well established to date. One study studied the utility of CoQ10 for patients with CPEO, MELAS and MERRF; while Medical Research Council index scores for muscle strength were significantly improved post-treatment, other improvements in clinical assessments such as muscle endurance, fatiguability during daily living and reductions in serum lactate levels did not reach statistical significance [42]. Similarly, a separate study administered high-dose CoQ10 to 30 patients with mitochondrial cytopathy over 60 days and found minor clinical improvements and decreased serum lactate, but did not improve muscle strength [43]. A key limitation of conventional oral CoQ10 is its poor bioavailability, arising from its large, highly lipophilic structure and correspondingly limited aqueous solubility [44]. Several strategies have been developed to address this. Idebenone, a short-chain synthetic CoQ10 analogue, offers improved aqueous solubility and blood–brain barrier penetration, and has demonstrated efficacy in LHON [45]. Liposomal and nanoparticle-based CoQ10 formulations have similarly been developed to improve gastrointestinal absorption and systemic bioavailability, for instance through crystal modification to increase particle surface-area-to-volume ratio, thereby enhancing dissolution and absorption [46]. Other nutritional supplements under investigation include thiamine, biotin, riboflavin and various other vitamins, cofactors and antioxidants [40].

Nucleoside bypass therapy: Oral deoxynucleoside substrates—deoxycytidine and deoxythymidine—are an emerging therapy for TK2 deficiency, by bypassing the deficient enzyme. An open-label study demonstrated better survival rates and motor function compared to untreated patients, with a favourable side-effect profile [47], and further clinical trials are underway (NCT04802707, NCT06754098) [48].

Elamipretide is a cardiolipin-targeting peptide that stabilises the inner mitochondrial membrane, thereby enhancing electron transport chain efficiency and reducing production of reactive oxygen species (ROS) [49]. Elamipretide has been studied in several diseases involving mitochondrial dysfunction; in the TAZPOWER trial performed on patients with Barth syndrome, patients treated with elamipretide were found to have sustained benefits in skeletal muscle strength, cardiac parameters and fatigue scores after its 168-week open-label extension period [50]. Phase 1–2 trials have also demonstrated improved exercise intolerance and walking distance in mitochondrial myopathy patients, though evidence is still weak [51].

Mitochondrial Transplantation and Cell-Based Therapies: Mitochondrial transplantation has emerged in recent years as a revolutionary strategy to replace dysfunctional mitochondria. Intercellular mitochondrial transfer is possible via either tunnelling nanotubes (TNTs) for shorter-range transfer, or extracellular vesicles (EVs) for longer-range transport [52]. Mitochondria-containing EVs derived from induced pluripotent stem cell-derived cardiomyocytes have been found to improve adenosine triphosphate (ATP) production and oxygen consumption in hypoxic cells, and improved left ventricular function in murine models [53]. This proof of concept has been tested in a Korean study which found that PN-101—mitochondria isolated from umbilical cord mesenchymal stem cells—improved clinical outcomes in nine adults with refractory polymyositis or dermatomyositis just 12 weeks after a single intravenous injection [53]. Together, these findings provide preliminary evidence supporting the feasibility and potential therapeutic utility of mitochondrial transplantation, although clinical evidence in primary mitochondrial disease remains limited.

Despite this encouraging early evidence, there are several concerns surrounding mitochondrial transplantation and its risks. Foremost, mitochondrial transplantation can provide support to the recipient but does not address the underlying genetic cause of mitochondrial dysfunction [54]. Clinical studies to date have predominantly explored autologous mitochondrial transplantation, which minimises immunological and genetic incompatibility. However, this approach may be unsuitable when the patient’s mitochondria harbour pathogenic mtDNA variants [54]. Conversely, allogeneic transplantation introduces other challenges including donor–recipient mtDNA heteroplasmy, mitochondrial incompatibility and potential immune or inflammatory responses [54,55].

Accordingly, translation to clinical use will require careful selection of both mitochondrial source and recipient. Donor mitochondria should undergo assessment of mtDNA integrity, pathogenic variants, and mitochondrial functional parameters, with genetic compatibility testing where appropriate [56]. Serial assessment of donor-derived mtDNA and mitochondrial function should be performed, together with surveillance for potential immune or inflammatory adverse events as well as progression of disease in affected organs [55,56]. However, the acceptable degree of donor-derived heteroplasmy and its long-term complications remain uncertain. Further longitudinal studies are required to establish the safety and therapeutic efficacy of mitochondrial transplantation before its adoption into routine clinical practice [55].

## 4. Conclusions

When evaluating a patient with chronic ptosis, clinicians should consider broad differentials even when initial clinical examination suggests a more common cause. Opportunistic muscle biopsy at the time of planned oculoplastic surgery can be diagnostically useful, though not necessarily definitive, and we propose that this approach be considered as a low-morbidity adjunct in patients with atypical or treatment-refractory presumed ocular myasthenia gravis. While histological findings can provide diagnostic clues and narrow the differential diagnosis when interpreted alongside the clinical picture, genetic testing remains necessary for a definitive diagnosis. Once mitochondrial disease is suspected or diagnosed, structured systemic evaluation should be performed. We recommend a conservative, staged approach to surgical correction; in our patient, this achieved a sustained improvement in ptosis with no lagophthalmos or exposure keratopathy at one-year review.

## Figures and Tables

**Table 1:** Timeline of key management events.

Date/Period	Clinical Findings	Management Plan and Results
June 2019	Brow ptosis, dermatochalasis, right exotropia (longstanding). No motility limitation, fatigability, or systemic MG features.	Referred to ophthalmology after abnormal DR screening. Impression: strabismic amblyopia
2019–2021	—	Annual follow-up
May 2022	Progressive ptosis noted. Positive enhanced Gorelick sign, Cogan’s lid twitch, and ice-pack test. Mild limitation of right downgaze with moderate limitation of left upgaze. No diplopia or fatigability was noted.	Oculoplastics assessment raised clinical suspicion for ocular MG, and patient was managed as possible seronegative ocular MG with a differential of aponeurotic ptosis. Trial of pyridostigmine (Mestinon) commenced.
	AChR and MuSK antibodies negative; RNS normal; sfEMG of orbicularis oculi showed a normal mean jitter (22.08 µs) but 2 of 12 individual fibre pairs exceeded the upper limit of normal (34 and 47 µs); neostigmine test not performed (bradycardia).	
2022–2025	Patient did not find pyridostigmine helpful, and reported no diurnal variation in symptoms.	Lost to follow-up
March 2025	Patient returned, keen for surgical correction of ptosis. Pre-operative MRD1 −1 mm (both eyes); palpebral aperture 2 mm (right), 3 mm (left); levator function 8 mm (right), 9 mm (left).	Counselled for staged procedure: direct brow lift, then upper eyelid blepharoplasty
1 July 2025	—	Direct brow lift performed. Incisional biopsy of right frontalis muscle taken opportunistically, given uncertain aetiology. Histopathology: scattered atrophic fibres with subsarcolemmal granularity/vacuolation; COX and SDH activity retained in the majority of fibres; GT without ragged-red fibres. Findings considered supportive of, but not diagnostic of, mitochondrial myopathy.
2 September 2025	Residual ptosis after brow lift.	Bilateral upper eyelid blepharoplasty performed
August 2024–July 2025	—	Cardiac evaluation in view of ischaemic heart disease and bradycardia: transthoracic echocardiogram (August 2024) showed preserved LV systolic function (EF 65%); myocardial perfusion imaging (September 2024) showed no ischaemia; 24 h Holter (July 2025) showed sinus bradycardia (mean rate 58/min) with infrequent ectopics and brief runs of atrial tachycardia, without high-grade conduction block. Audiology and fundoscopy unremarkable.
September 2026	No lagophthalmos or keratopathy. Post-operative MRD1 1 mm (right), 0 mm (left); palpebral aperture 7 mm (right), 6 mm (left); levator function 9 mm (right), 6 mm (left).	Genetic testing offered on multiple occasions; declined by the patient, most recently in August 2026

AChR: acetylcholine receptor; MuSK: muscle-specific kinase; RNS: repetitive nerve stimulation; sfEMG: single-fibre electromyography; MG: myasthenia gravis; DR: diabetic retinopathy; LV: left ventricular; EF: ejection fraction.

**Table 2:** Clinical features favouring myasthenia gravis versus mitochondrial disease.

Findings Favouring Myasthenia Gravis	Findings Favouring Mitochondrial Disease
Positive Cogan’s lid twitch sign	Symmetric, bilateral, slowly progressive ptosis
Enhanced Gorelick sign	Symmetric ophthalmoplegia with no diplopia despite ophthalmoparesis
Positive ice-pack test	No fatigability or diurnal variation reported at any visit
Borderline-increased jitter on sfEMG (equivocal)	Negative AChR and MuSK antibodies
	Normal repetitive nerve stimulation
	No symptomatic benefit from pyridostigmine trial
	Absence of systemic MG features (bulbar, limb, respiratory) throughout course
	Muscle biopsy: atrophic fibres with subsarcolemmal granularity and vacuolation

AChR: acetylcholine receptor; MuSK: muscle-specific kinase; RNS: repetitive nerve stimulation; sfEMG: single-fibre electromyography.

**Table 3:** Adult-onset mitochondrial diseases relevant to this patient’s differential.

Condition	Key Ocular Features	Other Clinical Features	Inheritance Pattern and Genetics	Notes	Ref.
Sporadic CPEO	Bilateral symmetric ptosis, progressive ophthalmoplegia, no diplopia; may progress to lagophthalmos/ectropion from orbicularis weakness	Usually isolated; mild proximal myopathy may occur	Sporadic, often occurs due to a single large-scale mtDNA deletion; commonly 4977 bp deletion	Most common cause of isolated adult CPEO; consider especially in the absence of a family history	[4,19,20]
Autosomal dominant PEO (adPEO)	Bilateral symmetric ptosis and ophthalmoplegia, similar to sporadic CPEO	Variable: may include peripheral neuropathy, ataxia, depression, parkinsonism, cataracts, or hypogonadism depending on the causative gene	Onset typically 20–50 years of age. Various known associated genes with pathogenic variants: POLG1, TWNK, OPA1, RRM2B, DNA2 etc.	Consider if a family history of PEO across successive generations is present	[21]
Autosomal recessive PEO (arPEO)	Bilateral symmetric ptosis and ophthalmoplegia, similar to sporadic CPEO	POLG-related disease may include sensory ataxic neuropathy and dysarthria (SANDO); MPV17-related disease has a milder adult phenotype than its infantile hepatocerebral form	Various known associated genes with pathogenic variants: POLG1, TWNK, C1QBP, TOP3A, LIG3, RNASEH1, TK2, TYMP etc.	Consider if consanguinity or an affected sibling is present	[21]
Late-onset CPEO-plus	Bilateral symmetric ptosis and ophthalmoplegia, similar to sporadic CPEO, with systemic features in later stages	One or more of: hearing loss, ataxia, neuropathy, parkinsonism, endocrinopathy	Variable (mtDNA deletion or nuclear gene). Overlaps with the genetics of sporadic CPEO, adPEO and arPEO above	Consider if new systemic features are detected, or if they emerge on follow-up	[22]

CPEO: chronic progressive external ophthalmoplegia; mtDNA: mitochondrial DNA.

## Data Availability

The data presented in this study are available on request from the corresponding author due to patient privacy reasons.
